# Does ‘sub-threshold’ ventilatory stress promote healing after lung injury?

**DOI:** 10.1186/s40635-024-00644-5

**Published:** 2024-07-02

**Authors:** John J. Marini, Rebecca L. Kummer, Patricia R. M. Rocco

**Affiliations:** 1 Department of Pulmonary and Critical Care Medicine, University of Minnesota, Minneapolis, St Paul, MN USA; 2 Laboratory of Pulmonary Investigation, Carlos Chagas Filho Biophysics Institute, Federal University of Rio de Janeiro, Rio de Janeiro, Brazil

## Abstract

Excessive tidal stretching may initiate damage or retard healing after lung injury. However, it is seldom considered whether intracycle power and ventilatory forces of lesser magnitude than those required to cross an injury threshold might stimulate or accelerate beneficial adaptive responses. Acute lung injury is a dynamic process that may exhibit phase-dependent reparative responses to mechanical stress broadly similar to physical training, body trauma or sepsis. We propose that lower stress may not always be better through all phases of ARDS; moderately high tidal airway pressures that stay below the threshold of global injury may have potential to speed healing of the injured lung.

## Lung injury and recovery

The repetitive mechanical forces of ventilation may be well-tolerated or produce injury by inflicting structural damage or stimulating inflammatory and remodeling responses. In the clinical setting, non-protective mechanical ventilation in patients with ARDS may lead to lung damage, causing ventilator-induced lung injury (VILI) [1]. With major outcomes from randomized clinical trials in mind, prudent medical practice currently restricts end-inspiratory plateau and driving pressures during passive ventilation to maximums of 30 and 15 cmH_2_O, respectively, whenever feasible to do so. It is generally accepted that repeated excessive tidal stretching above such stress/strain thresholds may initiate damage or retard healing after lung injury [1]. However, it is seldom considered (and certainly more controversial) whether intracycle power and ventilatory forces marginally less than those required to cross the injury threshold might stimulate beneficial adaptive or reparative responses. Because acute lung injury is a dynamic process with a relatively acute onset and a longer phase of healing, it may, in theory, exhibit phase-dependent reparative responses to mechanical stress broadly similar to other acute insults such as physical body trauma or sepsis [2].

For example, when evaluating repair processes of ARDS, it is interesting to consider a theoretical parallel between acutely overstrained skeletal muscle and lung tissues damaged by excessive stress and strain beyond their injury thresholds. During the phase of rehabilitation and recovery from injury of the musculoskeletal system, as well as when training for strenuous exercise activities during health, graded increases of stress are introduced to gradually build strength without incurring lasting damage [3].

Along this line, the pace as well as the amplitude of the high-stress ventilation may influence the tissue response that follows. In a series of short-term experiments performed in lung pre-injured rats, we have previously shown that the rapidity with which increments are made of tidal volume (V_T_) [4], PEEP [5], ventilation frequency [6], and recruitment maneuvers [6, 7] influences the degree and nature of the injury that results from exposure to the same maximal level and duration of high-stress ventilation. Such results suggest that adaptation to and tolerance of higher than baseline ventilatory stress may begin over a relatively short time interval. Over the long term, gradual remodeling after inflammatory injury is strongly influenced by moderate and high-stress mechanical forces [8, 9].

## Molecular contributors to lung repair

**Fig. 1:**
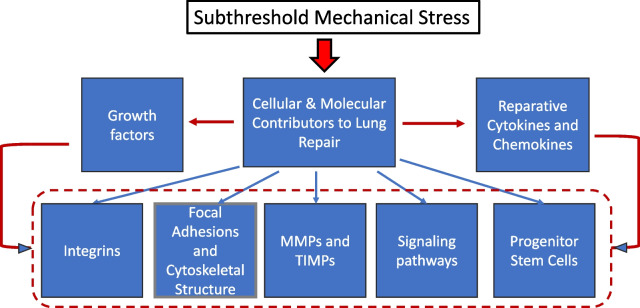
Possible influence of sub-threshold mechanical stress on the cellular and molecular contributors to lung tissue repair. These are disease stage dependent and expressed through direct, indirect, and interactive mechanisms

Blood flows may be either beneficial (‘nutritive’) or VILI-promoting [10]. Perfusion, together with the balance between proinflammatory and anti-inflammatory processes, determine the transformation of injured tissue over time. In lung injury, competing molecular pathways mediating both inflammation and cellular repair are set into motion from the onset of the initial insult, with the competitive relationship between them strongly influencing the extent of damage and rate of progression or recovery at any specific point [11]. Biomolecules that guide repair of tissue injury include growth factors [transforming growth factor (TGF)-b, epidermal growth factor (EGF), keratinocyte growth factor (KGF), and hepatocyte growth factor (HGB)], chemokines [monocyte chemoattractant protein-1 (MCP-1)] and C–C Motif Chemokine Ligand 2 (CCL2), interleukins (IL-1, IL-2, IL-4, IL-6, IL-13), and prostaglandins (PGE2) [11]. These factors, many of which are sensitive to mechanical stress, coordinate activities involving integrins, extracellular matrix components (fibronectin, collagen, laminin, elastin, proteoglycans and glycosaminoglycans), matrix metalloproteinases (MMP-1, MMP-7, MMP-9), tissue inhibitors of metalloproteinases (TIMPs), focal adhesions, and cytoskeletal structures to promote cell spreading and migration [12–14] (Fig. 1). Key signaling pathways susceptible to mechanical forces are important in regulating these processes, including Hippo/YAP, RhoA/ROCK, ERK1/2, JAK/STAT, and Wnt/beta-catenin pathways [14]. Progenitor stem cells (e.g., alveolar type II epithelial cells) recruited into the injured area may proliferate and phenotypically differentiate, leading toward recovery of epithelial function. On the other hand, sustained mechanically driven and dysregulated repair processes involving TGF-$\beta$ and epithelial–mesenchymal transition may promote lung fibrosis [15].

## Conceptual hypothesis

**Fig. 2:**
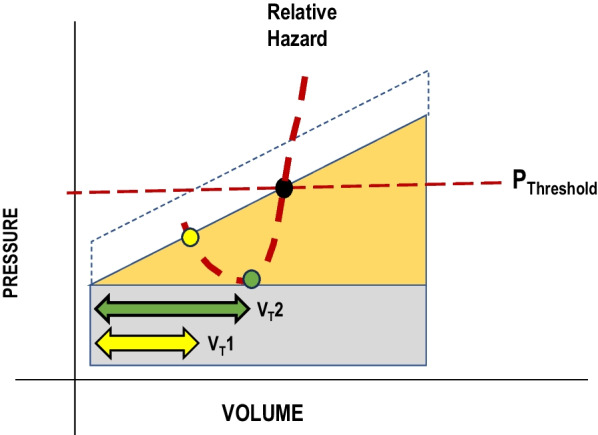
Inflation elastic energy (pressure–volume area) and relative injury hazard (dashed heavy line) at two tidal volumes (V_T_s) with PEEP unchanged. Relative hazard falls as tidal volume increases from a low initial value (V_T_ 1, yellow) to a higher but sub-injury threshold value (V_T_ 2, green). At still higher tidal volumes, this benefit fades and eventually reverses as stretch approaches and then crosses the average stress threshold for injury, as indexed by pressure recorded at the airway opening (P threshold)

Because the lung is rich in receptors that mediate cellular molecular responses to mechanical stress (e.g., integrins), we propose that modestly elevating tidal stresses and strains to approach but not exceed the injury threshold may not only improve perfusion but also shift the signaling balance from dysfunction toward reparation and more rapid recovery from acute lung injury (Fig. 2). Key to this hypothesis is the interstitial matrix, which contributes to structural integrity, mediates metabolic processes and, like skeletal muscle or tendon, has a fibrillar structure whose individual elements may either rupture when stretched beyond a strain threshold [16, 17] or, in theory, promote reparative signaling at lower levels of expansion or contraction of the lung unit. The vulnerability of lung tissue to high airway pressure (inversely related to its injury threshold) varies with its underlying metabolic environment, phase of progression or resolution of injury and the applied transpulmonary pressure. The latter is a function of lung region, body position and extent of diaphragmatic activity [18].

### Potential benefits of ‘sub-threshold’ stretch

Possible mechanisms of benefit from increased stretch include direct molecular signaling [19], enhanced surfactant renewal [20], and recruitment of collapsed lung units [21]. Stable recruitment, which may result from PEEP, increased V_T_ or upright body positioning, increases the number of aerated lung units that comprise the ‘baby lung’ of ARDS. Doing so lessens the number of stress-focused interfaces as well as the concentration of mechanical power [22]. Assuming that microvascular compression does not occur within distended airspaces, beneficial perfusion (nutritive blood flow) of stably recruited units may thereby be enhanced by reduced vasoconstriction in those now better oxygenated and less acidotic recovering lung regions [23, 24]. Prone positioning, which sustains gently increased traction quasi-selectively to non-dependent lung tissues, may accentuate such changes in dorsal zones of the lung [25]. In current clinical practice a wide range of V_T_s and PEEP levels that are accompanied by driving pressures < 15 cmH_2_O and plateau pressures < 30 cmH_2_O are considered safe and ‘lung protective’, regardless of disease stage [26–28]. It is conceivable that V_T_s and PEEP combinations at the higher end of this ‘safe stress’ range stimulate healing responses, especially after the initial phase of edema, collapse, and high vulnerability to mechanical stretch (Fig. 2). If so, within the same lung exposed to a given *airway* pressure, there exists a spectrum of transpulmonary pressures, raising the likelihood that some regions may be encouraged to heal faster than others.

## Key modulating factors

### Stretch timing

Establishing the right timing is crucial—early in the disease, the lung may be more vulnerable to stretching damage, while later, insufficient tidal stretch might impede the repair and remodeling process. Simply put, ‘lower stretch’ is not necessarily better at all injury stages. While we envision that the early stage is likely to be more vulnerable to damaging stress and mechanical power, these thresholds are largely unknown for a specific individual at any given time. That said, we think it likely that there may not only be tolerance of but reparative benefit from well-modulated (‘sub-threshold’) tidal stresses at each stage.

### Disease severity

In concept, all severities of injury have different global and regional thresholds for stress injury and strain tolerance. Our hypothesis would suggest that the relevant global injury threshold should not be crossed at any severity or stage.

### Respiratory rate

Regarding the role of respiratory rate (RR), in our view, RR assumes VILI importance only if ‘above-threshold’ tidal stress and strain criteria are satisfied as a precondition for the individual inflation cycle. Strong advocates of the ‘excess power causes VILI’ concept may not recognize a stress precondition for damage. In that unbridled and (we think) less likely scenario, a high enough RR that pushes close to (but does not exceed) a cumulative *energy* threshold for damage might also stimulate repair.

*Essential questions* regarding any proposed ‘moderated high stress’ benefit include those relating to its amplitude, timing, and patterning. These unexplored uncertainties are numerous, but all relate to the query: ‘Under which conditions can a mechanical stimulus or sequence of tidal stretch/recovery cycles boost repair processes without injuring?’ Because restoration of normal permeability may theoretically precede reversal of alveolar instability [29], time since injury onset may determine benefit or hazard from increased regional blood flow. When, therefore, can recruitment be accomplished without repair-impeding interstitial edema? In other words, what are the relative time courses of restored integrity of the leakage barrier and potential for recruitment? Whether any benefit of moderated high stress accrues to its sustained application (for example by PEEP or body positioning) or by its intermittent application (for example by raised V_T_) is undetermined. How is the injury threshold of applied pressure best defined and monitored at any given stage of injury resolution? Can levels of stress and strain that avoid net damage be guided by numerically defined pressure limits or by monitoring circulating biomarkers? Do shorter applications of airspace pressures that approach but do not cross the injury threshold, with each followed by a longer recovery phase of “lung protective” low V_T_ s and pressures, mimic conditioning exercise regimens of strenuous effort and rest?

## Summary

We emphasize that while the foregoing discussion may provide an intriguing and somewhat novel conceptual framework, it does not propose a pragmatic and actionable roadmap for modifying clinical behavior. Our largely unconfirmed but distinctly plausible hypothesis challenges current understanding of the timing, tolerance, and repair promotion of tidal stresses. It implies that personalization of ventilation may benefit from closer attention both to the extent of lung injury and the phase of recovery, which influence tolerance and consequences of tidal stretch. If so, lower inflation stress may not always be better, and fixed guidelines for V_T_, plateau pressure and driving pressures ideally need adjustment as tissue properties change over time. Indeed, moderately high tidal airway pressures that stay below the changing global stress threshold for injury may favor recruitment, restore generation of functional surfactant, improve perfusion to injured tissue, and speed the healing of the injured lung.

## Data Availability

Not applicable.
